# Dr. P. O. Hooper

**Published:** 1902-09

**Authors:** 


### OBITUARY NOTE.

Dr. P. O. Hooper, of Little Rock, Arkansas, died July 29, 1902, on a railway train en route to California, aged 68 years. His disease was alleged to be asthma. Dr. Hooper was vice-president of the American Medical Association and as such presided at the St. Paul meeting in 1882. He was an alienist of distinction and for many years was superintendent of the Arkansas state insane asylum.

